# Difficulties among Teachers’ Emotional Regulation: Analysis for the Development of Student Well-Being in Chilean Schools

**DOI:** 10.3390/bs14090749

**Published:** 2024-08-27

**Authors:** Gerardo Fuentes-Vilugrón, Eduardo Sandoval-Obando, Felipe Caamaño-Navarrete, Carlos Arriagada-Hernández, Paulo Etchegaray-Pezo, Flavio Muñoz-Troncoso, Isabel Cuadrado-Gordillo, Pablo del Val Martín, Enrique Riquelme-Mella

**Affiliations:** 1 Faculty of Education, Universidad Autónoma de Chile, Temuco 4780000, Chile; gerardo.fuentes@uautonoma.cl (G.F.-V.); paulo.etchegaray@uautonoma.cl (P.E.-P.); 2 Escuela de Psicología, Instituto Iberoamericano de Desarrollo Sostenible, Facultad de Ciencias Sociales y Humanidades, Universidad Autónoma de Chile, Temuco 4800916, Chile; eduardo.sandoval@uautonoma.cl; 3 Physical Education Career, Faculty of Education, Universidad Autónoma de Chile, Temuco 4780000, Chile; felipe.caamano@uautonoma.cl (F.C.-N.); carlos.arriagada@uautonoma.cl (C.A.-H.); 4 Grupo de Investigación Colaborativa para el Desarrollo Escolar (GICDE), Temuco 4780000, Chile; 5 Faculty of Social Sciences and Arts, Universidad Mayor, Temuco 4780000, Chile; 6 Department of Psychology and Anthropology, Faculty of Education and Psychology, University of Extremadura, 06071 Badajoz, Spain; cuadrado@unex.es; 7 Facultad de Educación y Ciencias Sociales, Observatorio Chileno de Educación Física y Deporte Escolar, Universidad Andres Bello, Las Condes, Santiago 7550000, Chile; pablo.delval@unab.cl; 8 Facultad de Educación, Universidad Católica de Temuco, Temuco 4810399, Chile

**Keywords:** emotional regulation, emotional dysregulation, teachers, emotional well-being, work fatigue

## Abstract

Introduction. Emotional regulation, a process that involves detecting and evaluating physiological signals in response to stressful events, is a crucial aspect of preparing students for school and ensuring teachers’ effectiveness, stress management, and job satisfaction. Method. This research, which adopted a quantitative approach, used a non-experimental comparative and cross-sectional design with a non-probabilistic sample by convenience. The study involved the participation of n = 1321 teachers (n = 125 preschool education; n = 645 primary education; n = 417 secondary education; n = 134 higher education). Results. The results revealed significant differences in the total scores of emotional regulation difficulty between teachers at the higher education level and primary and secondary school teachers, with the latter group showing higher levels of difficulty. Discussion. The findings suggest that the impact of emotional regulation difficulties affects professional performance, highlighting the importance of interventions aimed at improving teachers’ self-efficacy, resilience, and emotion regulation to reduce emotional exhaustion. Conclusion. From a practical point of view, our findings underline the importance of integrating emotional regulation training into pre-service teacher education and continuous teacher professional development programs. This could improve relational dynamics between students and teachers, fostering an environment conducive to teaching and learning processes.

## 1. Introduction

Historically, emotions have always played a role in all teaching and learning processes, mediating memories and giving meaning to the information processed by human beings [1]. These are crucial as they influence personal and others’ behaviors [2], impacting the socioemotional learning of teachers and students inside and outside the classroom [3]. This accounts for the importance of emotional regulation, which involves detecting and evaluating physiological signals before events that may or may not become stressful, leading to an emotional reaction [4,5]. The above strengthens the theoretical model proposed by Gross and Thompson, who state that emotional regulation is a goal-directed process that affects the intensity, duration, and type of emotion experienced [6,7]. In this sense, Gross and Thompson (2007) propose four phases of the emotional regulation process: (a) situation, as the way to choose or avoid activities based on the emotional impact that can be expected; (b) attention, which consists of identifying feelings according to the information to which we pay attention; (c) evaluation (cognitive change), which refers to the change in the way of thinking to change the way of feeling; and (d) response, which is the act of sharing and/or expressing emotion from an experiential, physiological, and/or behavioral perspective [8].

In the Chilean context, formal school-based instruction is a permanent process encompassing theoretical and intellectual aspects. It also aims to develop ethical, spiritual, values-based, artistic, physical, and emotional dimensions [9]. Emotional regulation processes have been studied in educational settings as they impact students’ engagement in learning experiences [10]. This teaching perspective helps learners to address learning challenges, making them better prepared to face academic and social obstacles [11,12]. The literature has already acknowledged and demonstrated how emotional components influence educational results at all academic levels [13]. For this reason, emotional regulation skills are fundamental for children’s school readiness and efficacy, stress, and job satisfaction [14,15]. 

In this regard, the teaching profession has become a demanding activity that involves constant emotional fluctuations [16]. However, there is an unspoken expectation that teachers should conceal negative emotions, such as frustration, sadness, and/or anger, and display emotions that are considered positive [16]. This aspect, combined with stress and excessive workload concerning available resources, can lead to emotional exhaustion [17,18]. This can negatively impact teaching performance, job satisfaction, emotional well-being, and student learning outcomes [19]. The close bond between teachers and students, as well as all interpersonal relationships within the school environment, mediates learners’ emotional regulation capacity [20,21,22]. In their study, Poulou and Denham demonstrated this perspective, emphasizing that teachers’ positive expressions of emotions significantly predicted students’ social and emotional competencies [23]. In essence, teachers need to understand and manage their emotional competencies to deploy them promptly when interacting with their students [24]. Equally, it has been demonstrated that emotion regulation can reduce emotional exhaustion syndromes in teachers. This means that the ability to recognize and regulate emotions and the capacity to make changes in the environment proactively can mediate the relationship between job strain and maladaptive emotional regulation practices [19].

Paradoxically, despite recognizing the importance of emotions in the Chilean educational context and in students’ learning processes, little work has been carried out in pre-service and in-service teacher education [25]. The teachers use two main strategies for emotional regulation: cognitive reappraisal, which involves consciously changing thoughts and behavior before the emotion fully emerges, and expressive suppression, which consists of regulating the outward expression of the emotion to prevent conflict [26,27,28,29,30]. However, the expressive suppression strategy is associated with higher levels of depressive symptoms, less success in mood recovery, and fewer interpersonal relationships [31]. In a context where knowledge of self-regulation strategies is lacking, along with the stress caused by teaching in a post-pandemic society, difficulties arise for emotional regulation. This construct is understood as the deficiency and/or inability to regulate the intensity and quality of emotions to generate an adequate emotional response and manage excitability, mood, and emotional overactivity [32].

Difficulties in emotional regulation can interfere with the well-being and functioning of individuals, which can manifest negatively in various ways: (a) biological aspects (substance abuse, self-harm, high blood pressure, increased heart rates, cancer, eating disorders, among others); (b) psychological and cognitive aspects (anxiety disorders, stress, depression, low self-esteem, among others); and (c) social aspects (attention deficit, aggressiveness, lack of empathy, ineffective communication, problems of coexistence, among others) [33,34]. Therefore, recognizing and understanding teachers’ emotional regulation strategies is crucial in educating children and young people. It could enable continuous improvement in the effectiveness and efficiency of teaching practices in the school context [35,36] and enhance relational dynamics between teachers and students. This element refers to the systematic interactions among educational agents in the classroom, which may lead to identifiable relational patterns [37,38]. Regulating emotional expression in teaching practices fosters an environment of emotional mimicry, where students automatically imitate each other’s nonverbal emotional behaviors [39]. Based on the above, the purpose of this research was to compare the levels of difficulties in the emotional regulation of Chilean teachers based on the educational level of the teaching institution that they worked in.

## 2. Materials and Methods

From a quantitative perspective, a non-experimental, comparative, and cross-sectional design was adopted. The researchers made this decision as they compared the emotional regulation difficulties of Chilean teachers who worked at the preschool, primary, secondary, and higher education levels.

### 2.1. Sample

The probabilistic convenience sample included 1321 male and female teachers (N = 266 men, 20.1% and N = 1055 women, 79.9%) who worked in Chile. The participants ranged in age from 22 to 71, and their years of work experience as teachers ranged from 0 to 50. As Table 1 shows, the highest participation was mainly among teachers working at the primary school level (N = 645, 48.8%), and the lowest participation was at the preschool level (N = 125, 9.46%).

### 2.2. Data Collection Instrument

The data collection instrument consisted of the Difficulties in Emotional Regulation Scale, which was adapted to Spanish for the Chilean population (DERS-E). This instrument consists of 25 items on a Likert scale (1 = rarely, 5 = almost always); the higher the score, the greater the difficulty in regulating emotions. The DERS-E is divided into five factors (Table 2), while the internal consistency, according to Cronbach’s alpha, reached 0.92 for the total instrument and varies between 0.67 to 0.90 for each factor of the emotions scale [40,41].

### 2.3. Analysis Procedure

Statistical analyses were performed using SPSS version 21.0 statistical software (SPSS Inc., Chicago, IL, USA). The Kolmogorov–Smirnov and Levene’s tests were used to evaluate the data’s normal distribution and the variances’ homogeneity. Continuous variables were expressed as means and standard deviation, and categorical variables as frequencies and percentages. Differences in the comparison of means between the two groups were performed using the Student’s *t*-test and Mann–Whitney U test as appropriate. One-factor ANOVA and Kruskal–Wallis tests were used to compare the means of more than two groups. In addition, Gabriel’s post hoc test was applied to establish differences between groups.

## 3. Results

In the dimensions of emotional dysregulation by gender, there were statistically significant differences in the total score (*p* < 0.001), emotional rejection (*p* < 0.001), daily interference (*p* < 0.001), emotional inattention (*p* < 0.023), emotional dyscontrol (*p* < 0.003), and emotional confusion (*p* < 0.0.45). In addition, women presented higher levels of difficulties in regulating their emotions and in the dimensions of rejection, interference, inattention, lack of control, and confusion (Table 3).

In the dimensions of emotional regulation by educational level where the participating teachers worked, there were statistically significant differences in the total score (*p* = 0.024) and the daily interference factor (*p* = 0.003). There were no statistically significant differences in the dimensions of emotional rejection (*p* = 0.061), emotional inattention (*p* = 0.095), emotional lack of control (*p* = 0.178), and emotional confusion (*p* = 0.090). Likewise, elementary school teachers presented higher levels of difficulty in emotional regulation (total score) (Table 4).

After conducting post hoc analysis to determine differences between groups, it was found that there are significant differences in total scores between primary education and higher education teachers (*p* = 0.009). There are also statistically significant differences between secondary and higher education teachers (*p* = 0.025) (Table 5).

Regarding the daily interference dimension, significant differences were found between primary and higher education teachers (*p* = 0.029) (Table 5).

## 4. Discussion

An in-depth study of the strategies and relational dynamics linked to emotional regulation by Chilean teachers provides relevant and current elements of analysis concerning their socio-pedagogical implications. A statistically significant finding of our study is the existence of difficulties with emotional regulation manifested by Chilean teachers according to gender. Specifically, women exhibited higher levels of difficulties in regulating their emotions in the dimensions of rejection, interference, inattention, lack of control, and confusion. Studies report that levels of regulation and differential emotional intelligence vary based on gender, race, and specialty and identify that men generally exhibit significantly higher levels than women [42,43,44]. From the perspective of these studies, women only obtain a higher score in valuing others’ emotions. On the other hand, a study on the implications of the pandemic reported a greater presence of self-care behaviors in men than in women [45]. 

In contrast to the above, it is crucial to make visible the multiple challenges that arose with the onset of the pandemic in Chile and the world, in which education professionals felt emotionally overwhelmed when facing a series of practical problems related to online teaching [46,47]. In this context, it has been identified that using emotion-focused coping strategies, which are more commonly employed by women, such as seeking emotional support and openly expressing emotions, may be more effective than a simple problem-solving approach, which men use more commonly [48]. Thus, women may have adapted better and felt more capable of coping with prolonged periods of confinement, thus perceiving fewer emotional problems [49].

Even though emotional regulation is installed as a multidimensional phenomenon [50], there are several possible explanations for the gender differences in emotional regulation difficulties observed among Chilean teachers, such as cultural differences in the processes of socialization and internalization of gender roles [51], as society often expects women to be more affectionate and expressive with their emotions. This scenario may lead them to internalize negative emotions such as frustration or anger, which could hinder their ability to regulate them effectively. In this sense, it has been observed that women are socialized to be mostly caregivers of others rather than recipients of care [52]. Precisely because of this, women may be able to direct compassion toward others in times of stress but not toward themselves [53]. Simultaneously, teaching often involves emotionally challenging work, as teachers must project a calm and positive demeanor toward students [54]. Women might face a more significant emotional workload due to social expectations, which could lead to greater difficulties in and out of the school classroom. In addition, female teachers might have less access to support systems within the school environment that help them to manage their emotions promptly [55,56].

On the other hand, it could be inferred that the stressful situations accumulated in the post-pandemic society and the various challenges that teachers face in the classroom may differ according to gender [57]. In other words, female teachers might face more microaggressions or disrespectful behaviors from students or parents, which generates greater emotional stress [58,59]. Therefore, differential analysis of emotional regulation from a gender perspective installs complex questions about the underlying factors contributing to these differences [60]. Future research could delve deeper into social, economic, and geographic (rural/urban) expectations, historical–cultural influences, and teachers’ coping strategies to unravel the complexities of emotional regulation in diverse educational settings. Similarly, they have manifested that female teachers report elevated stress levels stemming from student behavior and thus have greater sensitivity to external student behavioral problems than male teachers [61,62]. This is a cause for concern, especially considering that female teachers tend to make up the majority of primary and secondary schools [63,64].

Similarly, teachers are often exposed to different types of student misbehavior daily. As a result, they accumulate intense negative emotions that lead to emotional exhaustion and burnout [13], reaffirming the importance of further studies to explore these specific experiences. 

The results also showed significant variations in daily interference related to emotional regulation among teachers based on the educational level of their teaching institution. This finding suggests that the impact of emotional regulation difficulties extends beyond individual experiences and affects professional performance. Nevertheless, the results evidenced that primary education teachers reported higher levels of difficulty in emotional regulation compared to their higher education counterparts. These differences may be partly attributed to individuals becoming more proficient at regulating their emotions as they age [65]. This means that adults may be more inclined to regulate their emotions negatively, functionally modulating the potential impact of emotions experienced in their personal or professional context [66]. Likewise, such divergence is interesting as it underlines differential and heterogeneous challenges in teachers, depending on their different personal and professional development stages. Consequently, the results of this research partially coincide with those of a study with 1033 trainee teachers from various areas that found that the emotional intelligence levels of teachers with majors in Early Childhood Education and Science were higher than those of teachers with majors in Language, Social Studies, and Mathematics [67]. Precisely because of this, it could be pointed out that interventions aimed at improving emotional regulation skills should not only focus on individual coping strategies but also consider environmental factors and pedagogical–organizational support structures to mitigate interference and optimize the comprehensive well-being of teachers, regardless of the school level where they perform daily [68,69]. 

Similarly, other research highlights the importance of interventions to improve teachers’ self-efficacy, resilience, and emotion regulation to reduce emotional exhaustion [70,71]. In other words, teacher resilience mediates the association between emotion regulation and burnout [72,73,74]. On the other hand, studies have reported that engaging in potentially generative development could promote the emergence of resilient behaviors in teachers [75,76]. This practice may give them a clearer sense of purpose and greater flexibility in dealing with crisis processes, leading to higher subjective well-being. Undoubtedly, additional factors may also influence the relationship between these variables. This scenario highlights the complexity of the connection between these variables and emphasizes the necessity for further research in the field of educational sciences to identify other potential mediating factors.

The main limitation of the present study is its cross-sectional design. These variables should be measured in a longitudinal study to clarify the results better. Another limitation was that the measures of emotional regulation were obtained through a self-reported questionnaire, introducing a certain level of subjectivity in the results. Conversely, the study’s strength lies in the large sample size, which lends reliability to the results, and the diversity of participants from different regions of Chile.

## 5. Conclusions

In terms of the implications of the findings from this study for future research, several potential areas of exploration emerge. First, longitudinal studies that track the dynamics of emotional regulation among teachers over time could provide valuable information on the trajectory of these challenges and the effectiveness of intervention strategies in the short, medium, and long term [77]. This panorama would be enriched with mixed studies that delve into the lived experiences and coping mechanisms that teachers manifest in the classroom, systematizing the difficulties of emotional regulation for a comprehensive, evidence-based understanding.

This research highlights a clear challenge for Chilean education. Educational institutions should identify and understand the emotional burden that affects teachers’ work, promoting psychological support, peer support, and self-care opportunities. This approach would allow teachers to create healthy learning environments, maintain positive relationships with students, and respond effectively to classroom requirements. Furthermore, acknowledging the relevance of emotional regulation in education, particularly regarding the well-being of teachers and students, emphasizes the necessity of promoting ongoing training in this area. This would involve implementing professional development programs that enhance pre-service teacher education and providing in-service teachers with specific strategies and tools for managing their emotions and those of others.

From a practical point of view, these findings underscore the importance of integrating training in emotional regulation into initial teacher training and continuous in-service teacher development programs [77]. In this way, it could improve relational dynamics between students and teachers, fostering an environment conducive to teaching and learning processes. Similarly, organizational policies and support structures should prioritize teacher well-being, recognizing the impact of emotional regulation on professional satisfaction and retention rates. Finally, this research unveils the multifaceted nature of emotional regulation in Chilean teachers, highlighting gender disparities, educational level distinctions, and the implications of daily interference in pedagogical practice.

## Figures and Tables

**Table 1:** Distribution of the sample by level of education.

Level of Education	Number	Percentage
Preschool Education	125	9.46%
Primary Education	645	48.8%
Secondary Education	417	31.6%
Higher Education	134	10.1%
Total	1321	100%

**Table 2:** Factors of the DERS-E instrument.

Factor	Definition
Emotional rejection	Negative reaction to emotional responses from self and others.
Everyday interference	Emotions interfere with effective action toward a goal when people experience a negative emotion.
Emotional inattention	Difficulties recognizing and being aware of one’s emotions.
Emotional dyscontrol	Problems controlling one’s behavior when experiencing a high-intensity emotion.
Emotional confusion	Difficulties in differentiating emotions as they are experienced.

Source: excerpted from [33].

**Table 3:** Emotional dysregulation by gender.

Dimension	Gender	N	Mean	*p*
Total score	Male	266	56.92	
	Female	1055	63.46	<0.001 *
	Total	1321	62.14	
Rejection	Male	266	16.48	
	Female	1055	19.38	<0.001 *
	Total	1321	18.8	
Interference	Male	266	11.64	
	Female	1055	12.98	<0.001 *
	Total	1321	12.71	
Inattention	Male	266	10.85	
	Female	1055	11.54	0.023 *
	Total	1321	11.4	
Lack of control	Male	266	11.66	
	Female	1055	12.87	0.003 *
	Total	1321	12.63	
Confusion	Male	266	6.29	
	Female	1055	6.68	0.045 *
	Total	1321	6.6	

Note: * the difference in means is significant at the 0.05 level.

**Table 4:** Comparison by level.

Dimensions	Level	n	Mean	Standard Deviation	*p*
Total score	Preschool Education	125	62.28	19.603	0.024 *
	Primary Education	645	62.94	20.073	
	Secondary Education	417	62.59	21.293	
	Higher Education	134	56.81	18.508	
Rejection	Total	1321	62.14	20.331	
	Preschool Education	125	18.78	8.17	0.061
	Primary Education	645	19.13	8.385	
	Secondary Education	417	18.88	8.727	
	Higher Education	134	16.97	7.861	
	Total	1321	18.8	8.438	
Interference	Preschool Education	125	12.98	4.571	0.003 *
	Primary Education	645	12.91	4.389	
	Secondary Education	417	12.67	4.714	
	Higher Education	134	11.69	4.457	
	Total	1321	12.71	4.527	
Inattention	Preschool Education	125	11.56	4.701	0.095
	Primary Education	645	11.59	4.465	
	Secondary Education	417	11.35	4.395	
	Higher Education	134	10.49	4.037	
	Total	1321	11.40	4.431	
Lack of control	Preschool Education	125	12.25	5.73	0.178
	Primary Education	645	12.75	5.977	
	Secondary Education	417	12.89	6.103	
	Higher Education	134	11.58	5.225	
	Total	1321	12.63	5.929	
Confusion	Preschool Education	125	6.71	2.819	0.090
	Primary Education	645	6.57	2.762	
	Secondary Education	417	6.80	3.044	
	Higher Education	134	6.07	2.745	
	Total	1321	6.60	2.862	

Note: * the difference in means is significant at the 0.05 level.

**Table 5:** Post hoc between groups.

Dependent Variable	(I) NIV	(J) NIV	Difference in Means (I–J)	Standard Error	*p*
Total score	Preschool Education	Primary Education	−0.658	1.981	1
		Secondary Education	−0.31	2.067	1
		Higher Education	5.474	2.521	0.18
	Primary Education	Preschool Education	0.658	1.981	1
		Secondary Education	0.348	1.274	1
		Higher Education	6.132 *	1.925	0.009 *
	Secondary Education	Preschool Education	0.31	2.067	1
		Primary Education	−0.348	1.274	1
		Higher Education	5.784 *	2.013	0.025 *
	Higher Education	Preschool Education	−5.474	2.521	0.18
		Primary Education	−6.132 *	1.925	0.009 *
		Secondary Education	−5.784 *	2.013	0.025 *
Interference	Preschool Education	Primary Education	0.071	0.442	1
		Secondary Education	0.309	0.461	1
		Higher Education	1.282	0.562	0.136
	Primary Education	Preschool Education	−0.071	0.442	1
		Secondary Education	0.239	0.284	1
		Higher Education	1.211 *	0.429	0.029 *
	Secondary Education	Preschool Education	−0.309	0.461	1
		Primary Education	−0.239	0.284	1
		Higher Education	0.973	0.449	0.182
	Higher Education	Preschool Education	−1.282	0.562	0.136
		Primary Education	−1.211 *	0.429	0.029 *
		Secondary Education	−0.973	0.449	0.182

Note: * the difference in means is significant at the 0.05 level.

## Data Availability

Data are contained within the article.
